# A user-centred evaluation framework for the Sealife semantic web browsers

**DOI:** 10.1186/1471-2105-10-S10-S14

**Published:** 2009-10-01

**Authors:** Helen Oliver, Gayo Diallo, Ed de Quincey, Dimitra Alexopoulou, Bianca Habermann, Patty Kostkova, Michael Schroeder, Simon Jupp, Khaled Khelif, Robert Stevens, Gawesh Jawaheer, Gemma Madle

**Affiliations:** 1 City eHealth Research Centre, City University, London, EC1V 0HB UK; 2 Laboratory of Applied Computer Science – LISI/ENSMA, Chasseneuil, 86961 France; 3 Bioinformatics Group, Biotechnological Centre TU Dresden, Dresden, 01062 Germany; 4 Scionics Computer Innovation gmbh, Dresden, 01307 Germany; 5 School of Computer Science, University of Manchester, Manchester, M13 9PL UK; 6 Projet Edelweiss, INRIA Sophia Antipolis Méditerranée, Sophia Antipolis, 06902 France

**Keywords:** Evaluation Framework, Semantic Link, Online Evaluation, Health Protection Agency, Target Document

## Abstract

**Background:**

Semantically-enriched browsing has enhanced the browsing experience by providing contextualised dynamically generated Web content, and quicker access to searched-for information. However, adoption of Semantic Web technologies is limited and user perception from the non-IT domain sceptical. Furthermore, little attention has been given to evaluating semantic browsers with real users to demonstrate the enhancements and obtain valuable feedback. The Sealife project investigates semantic browsing and its application to the life science domain. Sealife's main objective is to develop the notion of context-based information integration by extending three existing Semantic Web browsers (SWBs) to link the existing Web to the eScience infrastructure.

**Methods:**

This paper describes a user-centred evaluation framework that was developed to evaluate the Sealife SWBs that elicited feedback on users' perceptions on ease of use and information findability. Three sources of data: i) web server logs; ii) user questionnaires; and iii) semi-structured interviews were analysed and comparisons made between each browser and a control system.

**Results:**

It was found that the evaluation framework used successfully elicited users' perceptions of the three distinct SWBs. The results indicate that the browser with the most mature and polished interface was rated higher for usability, and semantic links were used by the users of all three browsers.

**Conclusion:**

Confirmation or contradiction of our original hypotheses with relation to SWBs is detailed along with observations of implementation issues.

**Electronic supplementary material:**

The online version of this article (doi:10.1186/1471-2105-10-S10-S14) contains supplementary material, which is available to authorized users.

## Introduction

The sheer volume of resources available online makes it increasingly harder for users to find specific information and make quality judgements [1]. This problem is of particular concern to the life sciences, where sharing and making data available on the Web is widely accepted [2]. Commonly, scientists and medical practitioners need easy access to information about chemical compounds, biological systems, diseases, and the interactions between these entities, which requires this data to be effectively integrated [3]. The emerging Semantic Web (SW) technology [4] aims to provide a solution. While general purpose SWBs such as Tabulator [5] may enhance the search and browsing experiences of everyday users, SW technology in the life sciences has the potential to address the urgent needs of clinicians to find specific, quality-assured information under severe pressure of time [6]. Through SWBs, using underlying domain ontologies, context-based knowledge integration and semantically enhanced navigation can be achieved. A common assumption in the IT community is that the excitement about the SW technology will be shared by domain users. However, little attention has been given to evaluating SWBs with real users to demonstrate the enhancements and obtain valuable feedback.

The EU funded project Sealife [2] aims at providing easy access to disseminated information and resources in the life sciences' online databases. Its objective is the design and implementation of a semantic Grid browser to link the existing Web to the currently emerging eScience infrastructure. This has been accomplished using eScience's Web/Grid Services and its XML-based standards and ontologies. The main targets of Sealife are the infectious disease and molecular biology domains, illustrated respectively by the National Electronic Library of Infection (NeLI, http://www.neli.org.uk) portal in the United Kingdom, and the National Library of Medicine PubMed publications database http://www.ncbi.nlm.nih.gov/pubmed/ (accessible via GoPubMed technology).

To meet the objectives of the Sealife project, browsers have been implemented for different target audiences, including infectious disease clinicians (Group A1) and molecular biologists (Group A2). As each target group has different needs, prototypes have been developed following the principles of semantic browsing based on structured vocabularies or domain ontologies. To evaluate these distinct browsers, a common evaluation framework was needed.

We outline in this paper the work we have conducted to design a common evaluation framework for the Sealife SWBs and the hypotheses that were tested. While a Web browser navigates along links between documents, a SWB navigates along relationships in a web of concepts (Berners-Lee T, Hollenbach J, Lu K, Presbrey J, Prud'ommeaux E, Schraefel MC: **Tabulator Redux: Writing Into the Semantic Web**. Technical Report ECSIAMeprint14773. Electronics and Computer Science, University of Southampton; 2007). In this paper we use the term *Semantic Web Browser* (SWB) for any browser which: i) uses at least one knowledge organisation system (KOS), either a structured vocabulary or an ontology, to support the browsing; ii) is able to identify and highlight "useful" terms in the content being visited; iii) enables semantic interpretation of these Web pages and adds semantic hyperlinks to their highlighted terms, iv) gathers additional information from the highlighted terms, which may involve access to external data and services (e.g., European Bioinformatics Institute or PubMed) [7] called *targets*.

The rest of the paper is subdivided as follows. The introduction describes the 3 browsers of the Sealife project, providing the background for the evaluation methodology described in the Methods section. The introduction also outlines the aims and objectives of the evaluation framework and the hypotheses to be confirmed or contradicted by the evaluation process. Next we describe the results of the evaluation, which are discussed in the subsequent section.

This is the first user-centred evaluation of SWBs to be conducted using established, real-world Web resources as control platforms and recruiting participants from among the real-world users of these resources.

### The Sealife SWBs

To make the evaluation framework more comprehensible, we describe briefly in this section the different implementations of the 3 Sealife browsers. The first browser, COHSE-NeLI, is based on the Conceptual Open Hypermedia System (COHSE) [8] developed by the University of Southampton and the University of Manchester. The second is the CORESE-NeLI framework [7] based on the CORESE engine developed at INRIA. Finally, the GoPubMed/GoGene SWBs are developed at the Technical University of Dresden [9].

### The COHSE-NeLI SWB

**Figure 1:**
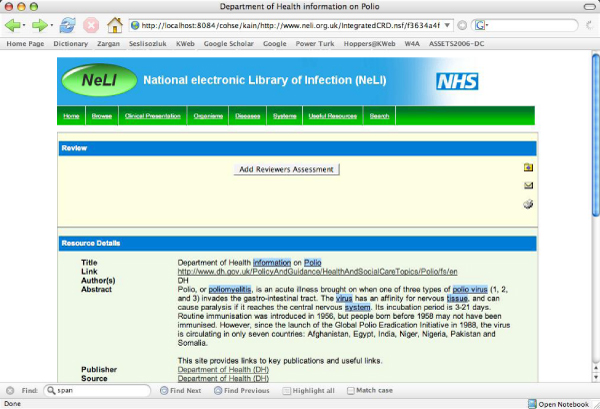
**COHSE semantic links as seen on the NeLI portal**.

**Figure 2:**
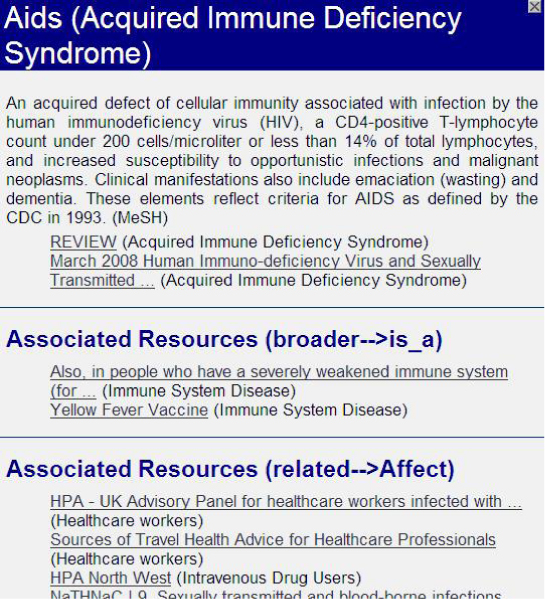
**COHSE semantic links: link boxes which appear after a click on the highlighted terms**.

The COHSE system [8] automatically adds hyperlinks on Web pages by recognising and highlighting terms contained in background knowledge, based on an ontology or KOS. (Figure 1). When a highlighted term is clicked, a link box appears (see Figure 2), populated with links to trusted external resources. For any highlighted term, resources are provided for broader, narrower, and related terms (e.g. affects/is_affected_by, is_symptom_of/has_symptom, causes/is_caused_by, treats/is_treated_by) obtained from the vocabulary. For the Sealife project, COHSE was adapted for the NeLI portal, and the version discussed in this paper uses the NeLI vocabulary [10] enriched with MeSH terms [11] as its KOS. The NeLI vocabulary formalises the infectious disease domain and is modelled in the SKOS language http://www.w3.org/TR/skos-reference/.

### The CORESE-NeLI SWB

The CORESE-NeLI [7] engine supports the navigation of a portal by the use of a knowledge artefact (either a structured vocabulary or a domain ontology). The browser can perform a) a semantic search and b) semantic browsing of the NeLI portal. The CORESE-NeLI engine bases its semantic search on semantic annotations generated from Web pages using the NeLI vocabulary, and using the relationships in the knowledge artefact (i.e., narrower, broader, related to) to retrieve annotated pages related to the user query. For semantic browsing, CORESE-NeLI can identify and highlight, in a Web page being visited, terms retrieved from a structured vocabulary. From the highlighted terms, it can then create links to related pages within the portal, enabling semantic browsing. A query can be built from the highlighted terms to query external resources such as Google and PubMed.

**Figure 3:**
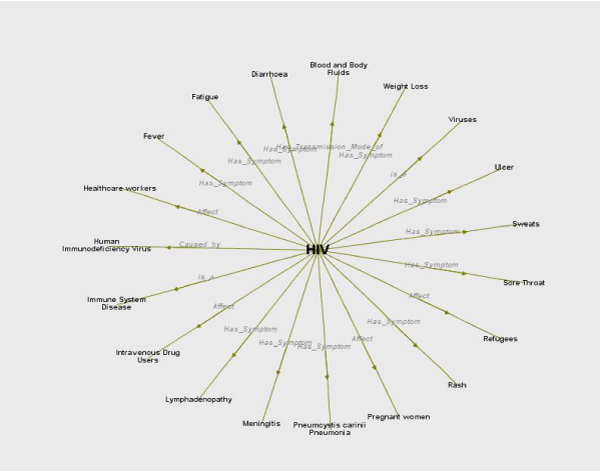
**The CORESE search box and graph showing terms related to "HIV" (Human Immunodeficiency Virus)**.

**Figure 4:**
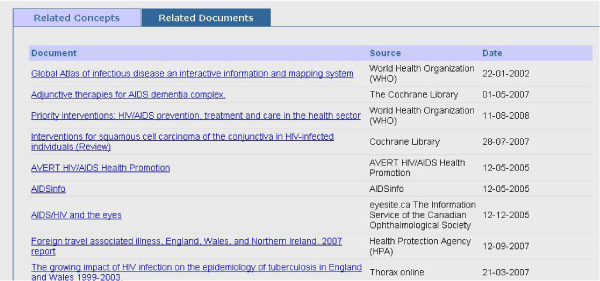
**CORESE-NeLI pane of related documents**.

CORESE is accessible via a plugin in Firefox. Entering a search term which exists in the NeLI vocabulary opens a tabbed pane. The first tab shows a graph of related concepts in the NeLI vocabulary (Figure 3), with which the user can navigate the NeLI Digital Library (DL) by double-clicking on a node or an edge. To the left of the graph is a history of recently visited search terms. The second tab shows a list of related documents (Figure 4).

### GoPubMed and GoGene for molecular biology

**Figure 5:**
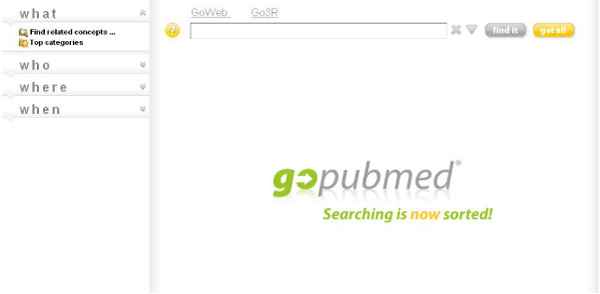
**The GoPubMed search portal**.

GoPubMed and GoGene are search technologies applied to the PubMed online database. GoPubMed (Figure 5) uses ontologies to deal with the wealth of medical and biological research literature by grouping literature by the underlying information in the abstract. GoPubMed offers name recognition and computational Web services. One of the major problems in text mining is the ambiguity of names of genes and proteins (especially crucial for computational Web services), as well as context-based terms used in molecular biology. GoPubMed and its underlying search engine handles this problem. As most scientists working in molecular biology have problems with finding the most useful and straightforward analysis tools for RNA or genomic sequences, through its computational Web services, the GoPubMed server aims to advance and streamline the process of sequence-based analysis.

**Figure 6:**
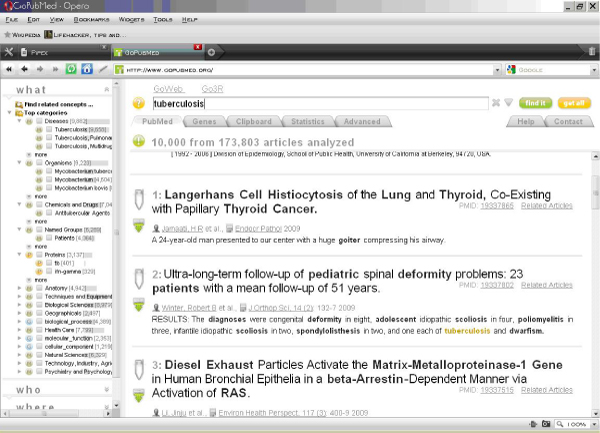
R**esults of a GoPubMed search for "tuberculosis"**.

A tree on the left of the screen categorises the results into "what" (subject matter), "who" (authors), "where" (geographical area), and "when" (date). The "what" category is further subdivided into "Top categories" (by number of results found). A search for "tuberculosis" yields results as shown in Figure 6.

### Aims and objectives of the evaluation

In the SW area in general, some comparable evaluations, raising interesting issues, have already been reported in the literature [12, 13]. The EON workshops initiative (International Workshops on Evaluation of Ontology-based tools) provides an environment for technical evaluation of SW tools. We focus in this paper on a user-centred evaluation of SWBs. The aim was to compare each SWB not to the other SWBs, but to a non-semantic control platform. The following hypotheses were made to test the key purposes of the SWBs: improving mobility and travel within the system and improving user satisfaction.

#### Mobility and travel within the system

**H1:** The SWB reduces the time taken for users to find information or perform tasks.

**H2:** The SWB shortens the pathway taken to find information or perform tasks.

**H3:** Where semantic links are available, users will always follow them instead of nonsemantic links.

#### User attitude and satisfaction

**H4:** Users find the SWB easier to use than the control platform.

**H5:** Where semantic links and ranking are available, users prefer them to non-semantic links and ranking.

**H6:** Use of the SWB is intuitive:

**a)** Users think the SWB helps them to find information or complete tasks.

**b)** Users intuitively understand how to use the SWB to find such information or complete tasks.

To prove or disprove the hypotheses, the following questions were considered:

#### Mobility and travel within the system

**O1:** time taken for users to find information or perform tasks

**O2:** pathway taken to find information or perform tasks

**O3:** use of semantic links compared with non-semantic links

**a)** Do users use semantic links?

**b)** Which semantic links are they using – tree, semantic relationships, etc.?

**c)** What percentages of links are non-semantic and semantic?

#### User attitude and satisfaction

**O4:** user satisfaction with the ease of use of the system

**O5:** user attitudes to the availability of semantic links and ranking

**O6:** user understanding of the SWB:

**a)** Does the user think it helps him/her find information or complete tasks?

**b)** Does the user understand how to use the SWB to find such information or complete such tasks?

The evaluation framework described in the Methods section was then designed to test these hypotheses and answer these questions.

Additional objectives were to ensure reusability of the evaluation process for future SWBs, and of the results for future development of the Sealife browsers.

## Methods

Because some Group A1 recruits were students, approval was obtained from City University's Ethics Committee. All student participants read an explanatory statement and signed a consent form before participating. All participants' privacy has been maintained and no personally identifiable data is included in any publication arising from the study. Group A2 did not require ethical approval.

### Sample populations

Table 1 shows the target population and the control platforms and intervention SWBs each population used. The initial aim was to recruit 10 users per intervention SWB.

**Table 1:** Sample populations for the evaluation

Population	A1 infectious disease practitioners	A2 molecular biologists
**Control**	NeLI	PubMed
**Intervention**	COHSE-NeLI	GoPubMed/GoGene
	CORESE-NeLI	

### Settings

The evaluation was carried out both online and in workshops. The online evaluation was necessary to evaluate the SWBs in real-world conditions, and to increase the number of participants. Because remote users' questionnaire answers may misrepresent their experience, their behaviour was tracked with Web server logs. The workshop evaluation was necessary to observe users' behaviour and collect further qualitative data with semi-structured interviews.

### Structure

Although users would become more familiar with the SWB by doing more tasks, and potentially give more accurate feedback, time constraints were recognised as a possible problem. To manage the risk that online users would fail to complete a lengthy evaluation, a short format, with fewer tasks, was devised for the online evaluation, and the long format, with more tasks, was used in the workshops. A complete list of tasks is provided in Additional file 1. Table 2 shows the final structure of the evaluation.

**Table 2:** Evaluation structure

Step	SC Users starting with the control platform	SI Users starting with the intervention SWB	
**1**	Pre-questionnaire regarding user demographics and previous experience with the control platform		Web server log collection
**2**	Task carried out using control platform	Task carried out using intervention SWB	
**3**	Post-task questionnaire		
**4**	Repeat steps 2 and 3 until half of the tasks are completed		
**5**	Task carried out using intervention SWB	Task carried out using control platform	
**6**	Post-task questionnaire		
**7**	Repeat steps 5 and 6 until all of the tasks are completed		
**8**	Post-questionnaire regarding user satisfaction and attitude		
**9**	Semi-structured interviews (workshops only)		

### Control/intervention split

Instead of splitting the users into a control group and an intervention group, the evaluation was structured so that each user would use both the control and the intervention systems. It was also decided that, for each respective SWB, all of the users would be given the same set of tasks to do in the same order. The split would be implemented through counterbalancing, with some users doing the first half of the tasks using the control platform and the second half of the tasks using the intervention SWBs, and other users vice versa.

### Data collection

Data collection from 3 sources was planned. The first source was the Web server logs collected automatically as users navigated the website. The second source was the pre-evaluation, post-task, and post-evaluation questionnaires as described in Table 2. The third source was the semi-structured interviews to be conducted at the workshops.

### Comparison with other evaluations

Hoeber and Yang [14] have identified a number of choices faced by designers of user evaluations for Web search interfaces.

#### Number of interfaces evaluated by each user

Because our goal was to compare the SWBs with non-semantic browsers rather than with each other, and because of anticipated time constraints on users, we chose a within-subjects rather than a between-subjects experiment design (exposing each user to both the control and intervention interfaces, rather than to just one interface). Because each intervention SWB was an enhancement to its control platform, a risk of bias typical of within-subjects experiments remained: users might apply knowledge of one interface to the next. This was handled by counterbalancing the order in which users were exposed to the control and intervention systems (see "Control/intervention split"). For the same reason we decided to use multiple tasks, rather than repetition of the same task.

#### Task definition

Another choice is between allowing users to choose their own search topics, or predefining tasks for them. We chose to predefine our own tasks because user-defined tasks would have made it difficult to define completion criteria. Sets of predefined tasks such as [15] are available, but not necessarily applicable to the biomedical domain nor to the features of our SWBs.

#### Uniformity of result sets

Ensuring that all of the SWBs provide access to the same result set [14] was not an issue for our study as the SWBs were being compared to non-semantic systems, rather than to each other. Whereas CORESE-NeLI retrieves results only from the NeLI DL, and GoPubMed/GoGene retrieves results only from PubMed, the purpose of COHSE is to provide external links, so result sets between control and intervention could not have been uniform for all SWBs.

#### Elicitation of relevance ratings

Rather than require users to rate the relevance of individual documents or rank their top results [16–18], we decided to use the post-questionnaire to capture subjective ratings of the overall relevance of results, and use the weblogs to record which documents were actually viewed.

#### Completion criteria

For Group A1, it was decided to have a single target document for each task, considered completed by the user's visiting that document. Because links on PubMed change frequently, there could be no specific target documents for Group A2, so the GoPubMed/GoGene task completion criterion would be the user's subjective perception of having found the answer. Asking participants to print out the results [17] would have been unfeasible, especially for COHSE's link boxes.

#### Time to completion

The weblogs would capture objective measures, and the post-task and post-evaluation questionnaires subjective perceptions, of time to completion.

#### Capturing responses to questionnaires

We chose Web rather than paper forms for the questionnaires, to accommodate remote users and to maintain participants' focus and facilitate data analysis. Verbal protocols were ruled out; measures of intuitiveness might also have been biased by users' overhearing each others' comments. It would have been unfeasible, and a distraction from the SWBs' functionality, to add features in the interface for capturing users' opinions [19].

### Implementation of data collection

Data was collected from the 3 planned sources. The Web server logs provided answers for O1, O2, and O3. The questionnaires provided answers for O4, O5, and O6a) and the interviews provided answers for O6b).

### Implementation of questionnaires

All of the evaluations began with a pre-questionnaire for demographic information (occupation/main degree, length of professional experience, preferred online research sources, experience of the control platform). Each task was followed by a post-task questionnaire containing 2 questions: *How well did the information you found answer the question?* (answer choices: Not at all, Partially, Fully) and *Was finding the answer in the information returned by the search engine:* (answer choices: Hard, Neither Hard nor Easy, Easy). Each evaluation ended with a post-questionnaire about ease of use of the system, information findability, relevance of information returned, overall system speed, and overall system likeability. Except for one question relating only to the SWB, each question required 2 answers: one for the control platform, and one for the intervention SWB [20]. An example is:

*I found the system unnecessarily complex* [21].

*a) Unmodified system (NeLI alone)*

*b) Modified system (NeLI + [SWB])*.

The answer choices were on Likert scales, commonly used in questionnaires to specify a level of agreement with a statement. An example would be a scale from 1 (strongly disagree) to 5 (strongly agree). Most of the answer choices for Group A1 were on a scale of 1 (worst) to 5 (best), and most of the answer choices for Group A2 were on a scale of 1 (worst) to 10 (best). Group A2 had additional questions about the functionality of GoPubMed/GoGene. The complete questionnaires are shown in Additional file 2.

### Implementation of semi-structured interviews

Workshop participants were interviewed where possible, using a loose structure with introductory questions (name, job title, etc.) followed by questions about the user experience such as *"What would make you want to use [the SWB] regularly?"*, a question which was worded to overcome reluctance to give negative feedback by reframing it as suggestions for improvement. The interview structure is shown in Additional file 3, notes from the interview in Additional files 4 and 5.

### Implementation of web server logs

The server logs of the respondents' actions were analysed using a combination of logs produced by the SWBs and the server at City University, which hosted the online evaluation questionnaire and the NeLI website. Each respondent was assigned a unique identifier (uID) at the start of the evaluation, which was then passed between each page of the online evaluation and the SWB and the NeLI or GoPubMed website.

### Implementation of tasks

The COHSE and the CORESE-based SWB tasks were defined by one of the evaluators, a lay person with no medical knowledge, and reviewed by a colleague with medical expertise. The goal was for the framework to be applicable to any SWB. While we believe that this goal has potentially been met by the framework as a whole, one part of the evaluation process cannot be generalised: the task definition. Since each of the SWBs was different in nature, the same tasks would not have been appropriate for each. COHSE uses the NeLI vocabulary to present links *external* to the NeLI DL. The CORESE-based SWB presents a graph of the vocabulary for navigation *within* the NeLI DL and sorts the search results according to the hierarchical position in the vocabulary of the relevant terms found in the documents. GoPubMed and GoGene are search technologies applied to the PubMed search engine.

For COHSE and the CORESE-based SWB, the tasks were counterbalanced with users with even-numbered uIDs starting with the intervention SWB, and those with odd-numbered uIDs starting with the control platform. Thus, the same task was sometimes answered with the SWB and sometimes with NeLI. The answer to each task was always located in a single target document. A task was considered complete when the user felt that the answer had been found, whereupon a "Completed" button on the task page took them to the post-task questions. Users were asked not to spend more than 5 minutes on any one question.

We were conscious of the contrivance inherent in posing questions the exact answer to which could only be found in a single target document. However, the need for authenticity had to be balanced against the need to know whether or not the test had been passed; detecting whether a single target document was found was the most unequivocal way to achieve that. The answers also needed to be detailed enough that participants would be unlikely to know every detail from memory, and so mark the question as answered without first searching for the answer. To counterbalance this contrivance, the questions needed to be general enough to be partially answerable through NeLI alone, and this had to be demonstrable in search results *without* a single specific target document.

For COHSE, the target document was only reachable through a prominently visible link in a link box. The link box would appear when the user clicked on a specific related term highlighted by COHSE and found either on the NeLI home page or after searching for relevant terms in the NeLI website. An example is ***What kind of certificate should be used for documenting yellow fever vaccination? Have there been any changes to the format in the past two years?***

For the CORESE-based SWB, the target document either could not be found using a search of the NeLI website alone (at least, not by using predictable search terms), or else the target could be found via NeLI alone, but ranked lower than 20 in the results. An example is ***What are the recommended guidelines for hygienic cleaning of surfaces after flooding?***

Dedicated tasks were also devised for GoPubMed (online) and GoGene and an extended GoPubMed (workshop). These were not counterbalanced but were in the sequence described in the paragraph "Format". They were designed to avoid, as much as possible, bias in favour of the SWB. Target documents were not specified for the GoPubMed evaluation because the results change on PubMed so frequently. The participants were told not to spend more than 8 minutes to answer each task. An example is ***What is the main role of the gene MMS2? Name 3 genes related to it in literature***(PubMed); and ***What other genes are related to Shh in literature? Name 3 of them***(GoGene). For the workshop, another PubMed task was ***Can you find any conserved domain information on Rab5?*** and an extended GoPubMed task was ***Can you find any conserved domain information on Apc11?***

### Incentives

As an incentive, all participants for all evaluations who chose to provide contact details were entered into a prize draw to win £100.00 in Amazon vouchers. As a minor incentive, and an opportunity for elicitation of further qualitative data through informal discussion, lunch was provided at all workshops. Where food could not be served, book or food vouchers were offered instead, and were cited by some interviewees as their motivation for attending. As time pressure increased, an additional incentive was added to each workshop session in the form of a prize draw for an iPod Shuffle, but whether this increased recruits' motivation is not clear.

## Results

### Recruitment of online participants

The online evaluation ran from December 2008 to March 2009. Users were recruited through advertisements and newsletter bulletins circulated to the mailing lists of NeLI and its companion site NRIC http://www.nric.org.uk, the National Resource for Infection Control), and news bulletins on the sites' home pages.

### Recruitment of workshop participants (Group A1)

The workshops for COHSE-NeLI and CORESE-NeLI (Group A1) took place in London, all at City University, except for one which was hosted by the Health Protection Agency (HPA) Centre for Infections. Recruitment was through invitations circulated to the NeLI and NRIC mailing lists, to the HPA, to the Infection Prevention Society, and to other organisations through the evaluators' professional contacts. The original plan was to hold one 2-hour workshop at a fixed date and time at City University followed by one hour for lunch and semi-structured interviews. However, due to the constraints on clinicians' time, acceptances were few and cancellations many. Because of these difficulties, a workshop was planned at the HPA Centre for Infections, where workstations were reserved in the library for staff to participate throughout the day. The event was advertised a week in advance, using posters and internal news systems; fliers and a stand were used in the canteen on the evaluation day. In this way, 14 participants were recruited, prompting the use of similar strategies at the two subsequent workshops that were held at City University.

### Recruitment of workshop participants (Group A2)

One workshop was held for GoGene and the extended GoPubMed (Group A2), at the Biotechnology Centre of the Technische Universität in Dresden, where postgraduate students constituted a source of real-world users. A successful recruitment strategy was through personal contacts of one of the evaluators, admittedly introducing some risk of bias, but securing attendance of a higher number of real-world users.

### Demographics

The following section describes the results for each SWB. Table 3 shows the number of participants from each target and non-target audience. Groups with a majority of participants from the target audience were the COHSE-NeLI online group, the CORESE-NeLI workshop group, and the GoGene/extended GoPubMed workshop group. Only 2 of the CORESE-NeLI online group completed any tasks, and one of those dropped out after the control tasks, leaving the intervention tasks untouched. A possible explanation is that CORESE requires installation of a plugin, which may have been off-putting to this user group.

**Table 3:** User demographics

SWB	Setting	# participants	Occupation	# participants
**COHSE-NeLI**	***Online***	***39***	Medical	21
			Scientific	6
			Other	12
	***Workshop***	***28***	Medical	4
			Information	10
			Student	14
**CORESE-NeLI**	***Online***	***4***	Medical	3
			Researcher	1
	***Workshop***	***14***	Medical	2
			Biological	6
			Information	3
			Unspecified	3
*Eliminated for completing tasks unrealistically quickly*				*2*
**GoPubMed**	**Online**	***141***	Biology	21
			Chemistry	1
			Physics	2
			Other	113
**GoGene/extended GoPubMed**	Workshop	***14***	Other	4 (of whom 3 scientists)
			Biology	8

### Format

All online evaluations were held in the *short format* of 4 tasks. For Group A1, the *long format* of 10 tasks for workshops proved too time-consuming and was abandoned in favour of the *short format*. For Group A2, the *long format* was used as planned, with 11 tasks instead of 10; 2 hours were allowed and proved sufficient. The tasks for Group A1 were counterbalanced as planned. The tasks for Group A2 were not counterbalanced: some tasks were answered with control only and some with the intervention SWB only. (See Additional file 1.)

### Objectives

#### O1: time taken for users to find information or perform tasks

The time taken per task was calculated from the online evaluation logs using the difference between task page and question page loading times. In the process it was noted that some users had not completed all the tasks, and others had completed all the tasks, but within an unrealistic timescale (e.g. more than 2 tasks completed under 60 seconds). These users were removed from the log evaluation. Additionally, logging was unavailable for the extended GoPubMed, so O1, O2, and O3 could not be answered for this SWB and log analysis of the extended GoPubMed is not included. Table 4 shows the average times spent using each system and the PubMed and NeLI websites. This suggests that GoPubMed tasks were the quickest in just over 2 minutes. The slowest tasks were for COHSE in just under 8 minutes.

**Table 4:** Average time for all tasks on each system in seconds

GoPubMed	GoGene	COHSE	CORESE	PubMed	NeLI
126	229	478	266	194	387

#### O2: pathway taken to find information or perform tasks

For the COHSE evaluation, none of the 28 users included in the log analysis for the short format found the target documents via COHSE.

For the CORESE evaluation, 11 users were included in the log analysis, of whom 8 started with CORESE and 3 with NeLI. Table 5 shows the number of users who viewed the target documents via the CORESE-based SWB. This shows that there were very few users who actually found the target documents with the CORESE-based SWB.

**Table 5:** Proportion of users who viewed the target document for each task

Task 1	Task 2	Task 3	Task 4
2/8	2/8	1/3	2/3

As stated, for the GoPubMed/GoGene there were no specific target documents for these SWBs. Logs of users' actions were however recorded to show how much a user was interacting with the site during the tasks. Users performed up to 40 actions whilst looking for the information and the majority of respondents used less than 15 actions to find the information on GoPubMed and less than 25 on GoGene. Access to the server logs for PubMed was not available for this evaluation.

#### O3: use of semantic links compared with non-semantic links

For COHSE, an indication of the use of semantic links is the number of times a highlighted term is clicked and the link box activated. A further indication is the number of views of external sites via COHSE. 6 users did not click on any highlighted terms and therefore did not use any of the semantic features.

In the short format, 132 sites external to NeLI were viewed from 97 link box activations. The largest number of link box activations per user was 15, the lowest 1; the median was 4 and the mode, 3.

Of those users who viewed external pages via COHSE-NeLI, the largest number of views per user was 42 for the short format and 192 for the long. The lowest for the short format was 1; for the long 24; the median was 5.5 for the short format, 82.5 for the long. The mode for the short format was 1; the long format had no mode. For CORESE-NeLI it was not possible to directly compare the use of semantic links with non-semantic links because all of the links that a user interacts with on the CORESE-based SWB can be classed as semantic. There were however 325 searches via the CORESE-based SWB compared to 91 searches via NeLI, suggesting that users interacted with the CORESE-based SWB more than they would a standard website. For GoPubMed, around 46% of users used the semantic features at least once, but as an overall percentage of activity, semantic activity was relatively low.

For GoGene, from a total of 270 recorded actions, 73 were classed as semantic actions (27%), generated by 10 individual users(one user was not found in the logs).

#### O4: user satisfaction with the ease of use of the system

##### Usability

COHSE scored 1 point higher (on a scale of 1 = worst to 5 = best) than control for complexity, the CORESE-based SWB 1 point lower, GoPubMed/GoGene the same. COHSE also scored as 2 points (out of 5) more satisfying than the control platform. The CORESE-based SWB scored worse than control for rigidity.

GoPubMed/GoGene scored 3 points higher (on a scale of 1 = hardest to 10 = easiest) than control for ease of use; there was no difference for COHSE and the CORESE-based SWB. GoPubMed scored 1 point higher (out of 10) than control for provision of help, with no equivalent question for the other SWBs.

##### Overall likeability of the system

COHSE scored better than control in 1 of the 3 questions posed (*I think that I would like to use this system frequently*), while the CORESE-based SWB scored worse than control for the same question, and GoPubMed/GoGene scored 3 points higher than PubMed (out of 10), a greater difference than the equivalent superior score for COHSE.

##### Overall system speed

COHSE scored worse than control for speed. GoPubMed and GoGene scored the same as control.

##### GoPubMed/GoGene functionality

Though there are no equivalent questions for the other SWBs, the functionality of GoPubMed and GoGene was well regarded (Table 6).

**Table 6:** Mode Scores for GoPubMed/GoGene functionality (Yes/No)

	Did you find the highlighting of ontology terms helpful? (Yes/No)	Did you get an overview over your search results from the tree on the left? (Yes/No)	Did you manage to navigate efficiently through the tree? (Yes/No)	Did you find any papers you would probably have missed with PubMed? (Yes/No)
**Mode**	Yes	Yes	Yes	Yes

#### O5: user attitudes to the availability of semantic links and ranking

COHSE and the CORESE-based SWB both scored better than control for absence of irrelevant results. The CORESE-based SWB scored better than control for relevance of results, while COHSE scored the same. GoPubMed/GoGene also scored better (by 3 points on a scale of 1 = worst to 10 = best) in the equivalent measures to those in which COHSE and the CORESE-based SWB triumphed. While COHSE scored best for absence of irrelevant results, GoPubMed/GoGene scored better than the CORESE-based SWB in this respect. GoPubMed/GoGene had the best scores for relevance of results.

#### O6: user understanding of the SWB

##### a) Does the user think it helps him/her find information or complete tasks?

The CORESE-based SWB scored 3 points worse (out of 5) than control for ease of finding answers (Table 7), for which GoPubMed/GoGene scored 2 points better (out of 10) than control, and 3 points better for speed of finding answers (Table 8).

**Table 7:** Findability of COHSE and the CORESE-based SWB: mode differences (scale 1 bad – 5 good)

	Speed of finding answers in info returned: Was COHSE or CORESE rated higher or lower than NeLI?	Ease of finding answers in info returned: Was COHSE or CORESE rated higher or lower than NeLI?
COHSE	0	0
CORESE	0	-3

**Table 8:** Findability of GoPubMed/GoGene: mode differences (scale 1 bad – 5 good)

	Speed of finding answers in info returned: Was GoPubMed/GoGene rated higher or lower than PubMed?	Ease of finding answers in info returned: Was GoPubMed/GoGene rated higher or lower than PubMed?
**Mode**	2	3

##### b) Does the user understand how to use the SWB to find such information or complete such tasks?

To test intuitiveness, all of the online evaluations, and the early Group A1 workshops, opened with minimal introduction. It quickly emerged that many users could not tell the control and intervention systems apart, giving detailed feedback on NeLI while assuming that the COHSE link boxes were advertisements or error messages. Consequently, introductory presentations were shown to each user at subsequent workshops. This reduced confusion, but users still said more introduction was needed. Even users who could tell NeLI apart from the SWBs complained of distraction by the NeLI website's user interface. A widely familiar control platform such as Google would have increased the contrast and foregrounded the benefits of COHSE in particular.

Users did not grasp the nature of the CORESE-based SWB at all, assuming it to be a keyword search with a graph attached. A detailed introduction would probably have greatly improved users' opinions.

The GoPubMed workshop opened with a 20-minute introductory lecture, and PubMed is a widely familiar system to use as a control. The difficulties encountered by Group A2 were noted as being generally more trivial than those found by Group A1.

### Overall post-questionnaire scores

In no case did GoPubMed/GoGene receive *worse* mode scores than control, whereas COHSE and the CORESE-based SWB received several lesser modal scores.

## Discussion

### Evaluation of the evaluation framework

For the GoPubMed SWB a number of the hypotheses formulated at the start of the evaluation were confirmed, especially regarding ease of use. For the other two SWBs, most of the hypotheses were contradicted. Table 9 shows how user feedback from each system agreed or disagreed with the hypotheses.

**Table 9:** Confirmation or contradiction of original hypotheses

	Hypothesis	COHSE	CORESE	GoPubMed
H1	The SWB reduces the time taken for users to find information or perform tasks.	No	Yes	No
H2	The SWB shortens the pathway taken to find information or perform tasks.	No (targets not found)	No (targets found by few users)	PubMed data not available for comparison
H3	Where semantic links are available, users will always follow them instead of nonsemantic links.	No	Yes	No
H4	Users find the SWB easier to use than the control platform.	Yes and No	No	Yes
H5	Where semantic links and ranking are available, users prefer them to non-semantic links and ranking.	Yes	Yes	Yes
H6	Use of the SWB is intuitive: **a)** Users think the SWB helps them to find information or complete tasks.	No	No	Yes
	**b)** Users intuitively understand how to use the SWB to find such information or complete tasks.	No	No	Yes

The evaluation study demonstrated that the evaluation framework is suitable for eliciting user perceptions of SWBs. The results have allowed us to answer our initial hypotheses fully for each SWB even though each SWB had a distinct implementation and used different aspects of the SW technology.

### Lessons learned

The strongest message received from the users was that a polished, mature, and friendly interface is indispensable for a positive user experience. Users cannot be expected to see through weaknesses in the interface and appreciate the underlying technical advantages. A related fact was the compatibility limitations of the Group A1 SWBs. While GoPubMed/GoGene work with any browser, COHSE works only with Internet Explorer 7 (many of the respondents use version 6) and the CORESE-based SWB only with Firefox. This presents an immediate barrier, particularly for novice users.

## Conclusion

A new evaluation framework for SWBs was designed and tested on 3 intervention SWBs, with participants recruited from the intervention systems' real-world target audiences. The control platforms were live, real-world systems with substantial numbers of existing users. Using this new evaluation framework, all of our initial hypotheses were successfully confirmed or contradicted (Table 9).

Overall, the framework successfully elicited a range of feedback on 3 distinct SW technologies. It was found that, although potentially easier to elicit feedback via online questionnaires, observing respondents in a workshop setting provides an excellent opportunity to gather both quantitative and qualitative data from larger numbers of users.

The evaluation showed that users tended to prefer the system (GoPubMed) that had the most mature interface, but were able to use the semantic features of all systems regardless of the interface or types of semantic links presented. For one of the browsers (CORESE) less time was taken to complete tasks compared to the control system. This, however, needs further investigation because few users actually found the target documents for Group A1.

The evaluation feedback will contribute directly to future versions of each SWB and there will be further analysis of the weblogs to determine the specific types of semantic links that were or were not used. We intend to use this evaluation framework further in evaluating other semantic technologies.

## Electronic supplementary material

Additional file 1: **Tasks**. all the evaluation tasks for Group A1 and Group A2. (TXT 15 KB)

Additional file 2: **Questionnaires**. all the pre-questionnaires, post-task questionnaires, and post-questionnaires for both Group A1 and Group A2. (TXT 12 KB)

Additional file 3: **Semi-structured interviews**. the questions given to all the interviewers to guide the semi-structured interviews. (TXT 403 bytes)

Additional file 4: **Appendix D: notes from semi-structured interviews, Group A1**. notes from all the semi-structured interviews for Group A1. (TXT 15 KB)

Additional file 5: **Appendix E: notes from semi-structured interviews, Group A2**. notes from all the semi-structured interviews for Group A2. (TXT 10 KB)

## List of abbreviations used

COHSE: Conceptual Open Hypermedia Service
CORESE: Conceptual Resource Search Engine
DL: Digital Library
ENSMA: École Nationale Supérieur de Mécanique et d'Aérotechnique
EU: European Union
HPA: Health Protection Agency
INRIA: Institut national de recherche en informatique et en automatique
IT: Information Technology
KOS: Knowledge Organisation System
LISI: Laboratoire d'Informatique Scientifique et Industrielle
MeSH: Medical Subject Headings
NeLI: National electronic Library of Infection
NRIC: National Resource for Infection Control
RNA: Ribonucleic Acid
SKOS: Simple Knowledge Organization System
SW: Semantic Web
SWAT4LS: Semantic Web Applications and Tools for Life Sciences
SWB: Semantic Web Browser
uID: unique identifier
XML: Extensible Markup Language.
